# Depression, anxiety, and stress mediate the associations between internet gaming disorder, insomnia, and quality of life during the COVID-19 outbreak

**DOI:** 10.1016/j.abrep.2020.100307

**Published:** 2020-10-22

**Authors:** Sara Fazeli, Isa Mohammadi Zeidi, Chung-Ying Lin, Peyman Namdar, Mark D. Griffiths, Daniel Kwasi Ahorsu, Amir H. Pakpour

**Affiliations:** a Student Research Committee, Qazvin University of Medical Sciences, Qazvin, Iran; b Social Determinants of Health Research Center, Research Institute for Prevention of Non-Communicable Diseases, Qazvin University of Medical Sciences, Qazvin, Iran; c Institute of Allied Health Sciences, College of Medicine, National Cheng Kung University, Tainan, Taiwan; d Psychology Department, Nottingham Trent University, Nottingham, UK; e Department of Rehabilitation Sciences, Faculty of Health & Social Sciences, The Hong Kong Polytechnic University, Hung Hom, Hong Kong, China; f Department of Nursing, School of Health and Welfare, Jönköping University, Jönköping, Sweden

**Keywords:** Internet gaming disorder, Insomnia, Depression, Anxiety, Stress, Quality of life, Adolescence

## Abstract

**Background:**

For many individuals, the COVID-19 outbreak has increased their psychological distress, changed their behaviors, and impacted their health. With more time spent indoors, many individuals have engaged in increased videogame playing. However, the associations between such behaviors during the COVID-19 outbreak period is unclear.

**Objective:**

The present study examined the mediating role of psychological distress (depression, anxiety, and stress) in the association between internet gaming disorder (IGD) and two health outcomes (insomnia quality of life) among adolescents during this COVID-19 pandemic.

**Methods:**

A cross-sectional study comprising adolescents (*N* = 1512) aged 13–18 years (mean age = 15.51 years) was utilized to assess measures on insomnia, depression, anxiety, and stress, IGD, and quality of life during the COVID-19 pandemic.

**Results:**

There were small to large significant relationships between the variables. Psychological distress (i.e., depression, anxiety, and stress) served as a strong mediator in the association between IGD and insomnia and quality of life. IGD directly influenced insomnia and quality of life among the participants.

**Conclusions:**

IGD is associated with different psychosocial outcomes comprising multiple pathways. Parents need to pay special attention to how much time and how frequently their children play videogames. Parents may need to assist their children in coping with psychological distress during the ongoing COVID-19 pandemic period.

## Highlights

• Depression, anxiety, and stress mediates the association between internet gaming disorder and insomnia.
• Depression, anxiety, and stress mediates the association between internet gaming disorder and quality of life.
• Internet gaming disorder may have multiple pathways in affecting adolescents’ sleep and quality of life.

## 1 Introduction

The coronavirus disease 2019 (COVID-19) pandemic has had a worldwide impact with over 25.1 million confirmed cases and over 844,000 deaths in 216 countries as of 31 August 2020 (World Health Organisation [WHO], 2020). This has negatively impacted on the economies (Gössling et al., 2020, Nicola et al., 2020), social relationships (Balanzá–Martínez et al., 2020, Chen et al., 2020a), and health (Ahorsu et al., 2020a, Choi et al., 2020, Lin, 2020) of individuals worldwide. To deal with the pandemic, governments of different countries are utilizing physical distancing, lockdowns, use of face masks and/or washing of hands as interim measures to deal with the spread of the virus due to the absence of cure or vaccine (Amin et al., 2020, Ko and Yen, 2020). However, interim actions such as lockdowns, physical distancing, and quarantining have reportedly led to heightened fears, stress, and anxiety among individuals worldwide (Ahorsu et al., 2020b, Amin et al., 2020, King et al., 2020, Ko and Yen, 2020, Lin, 2020). Therefore, individuals have taken solace in indoor activities which have the elements of social community and competition, including online gaming.

Online gaming can improve individuals’ health. Empirical evidence of gaming’s positive effects has been demonstrated for children undergoing chemotherapy, receiving psychotherapy (anxiety and depression), and having emotional and behavioral problems (Griffiths et al., 2017). Previous studies have also reported an improvement in cognitive skills such as reasoning, spatial awareness, and problem-solving after playing videogames (Hisam et al., 2018, Nuyens et al., 2019, Özçetin et al., 2019). Despite gaming’s aforementioned benefits, gaming can have its negative effects among some individuals if it is used excessively. Previous studies indicate that there is an association between problematic gaming behavior and health-related outcomes such as psychological health (e.g., positive association with depression and anxiety), social health (e.g., positive association with social phobia and loneliness), and physical health (e.g., decreased levels of physical activity, poorer sleep quality, increased hand and wrist pain) (Alimoradi et al., 2019, Männikkö et al., 2020, Wong et al., 2020, Wong and Lam, 2016). These studies demonstrate that the benefits of gaming are highly dependent upon the frequency and duration of videogame use. They also suggest that an individual should have good control over when and how much time they invest in gaming so as not to develop problematic (i.e., addictive) behavior. In extreme cases, internet gaming disorder (IGD) can occur and is more prevalent among adolescents and emerging adults.

Among adolescents, IGD is positively associated with psychological distress (depression, anxiety, and stress) and sleep quality (e.g., insomnia, sleep duration, and sleep latency) (Sosso and Kuss, 2018, Strong et al., 2018, Wong et al., 2020) while an inverse association has been reported between psychiatric distress (depression, anxiety, and stress) and quality of life (Adib-Hajbaghery et al., 2015, Freire and Ferreira, 2018, Raknes et al., 2017). Also, IGD has been found to be inversely associated with quality of life (Wartberg et al., 2017). Despite these inter-variable associations between IGD, psychological distress, insomnia and quality of life, the mechanisms underlying these associations have not been investigated during the COVID-19 outbreak period.

Furthermore, other studies have reported psychological distress (depression, anxiety, and stress) as mediators in relationships concerning quality of life (Bonsu et al., 2019, Hsu et al., 2014) which sits well in the present study given the findings of previous studies (Freire and Ferreira, 2018, Lin et al., 2018, Wartberg et al., 2017, Wong et al., 2020). Therefore, the present study examined the mediating role of psychological distress (i.e., depression, anxiety, and stress) in the association between IGD and insomnia, and IGD and quality of life among adolescents during the COVID-19 pandemic.

## 2 Methods

### 2.1 Participants and procedure

The sample comprised adolescents aged 13–18 years from 25 high schools in Qazvin (Iran) recruited between 22 May (2020) and 26 August (2020). The data were collected using a web-based self-report survey. An online consent form with study aims and information was sent to specific social media of these schools. Adolescents were eligible for the study if they: (i) were aged between 13 and 18 years (mean age = 15.51 years; SD ± 2.75) and (ii) agreed to participate in the study. Of the 2031 adolescents in the schools, 1512 adolescents completed online consent form (74.4% response rate). All adolescents (and their parents) gave online informed consent to participate in the study. The study was approved by the Ethics Committee of Qazvin University of Medical Sciences (no. IR.QUMS.REC.1399.103) and the Organization for Education in Qazvin.

### 2.2 Measures

#### 2.2.1 Demographic characteristics

A background information sheet was used to gather demographic data including age, gender, time spent playing online videogames, and educational level of the participants’ parents.

#### 2.2.2 Internet Gaming Disorder Scale-Short Form (IGDS9-SF)

The IGDS9-SF, developed by Pontes and Griffiths (2015), is a nine-item short self-report scale that assesses IGD according to DSM-5 criteria (American Psychiatric Association, 2013). Responses are rated on a five-point Likert-scale ranging from 1 (*never*) to 5 (*very often*). A higher score of the summed total indicates a greater degree of IGD. It has demonstrated very good psychometric properties in different languages (Monacis et al., 2016, Pontes and Griffiths, 2015, Pontes et al., 2016, Pontes et al., 2017) and specifically Persian (Wu et al., 2017), the version used in the present study with a Cronbach’s α of 0.90.

#### 2.2.3 Depression, Anxiety, and Stress Scale-21 (DASS-21)

The DASS-21, developed by Lovibond and Lovibond (1995), assesses psychological distress. More specifically, it assesses depression, anxiety, and stress with seven items for each subscale. All 21 items are rated on a four-point Likert scale ranging from 0 (*did not apply to me at all, never*) to 3 (*applied to me very much, or most of the time, almost always*) with a total score (sum of each subscale item score) for each subscale ranging between 0 and 21. The higher the DASS score, the higher the level of that corresponding subscale. The Persian DASS-21 version has reported having a very good to excellent internal consistency (Cronbach’s α = 0.84 to 0.91; Asghari et al. (2008) and among adolescents (Shaw et al., 2017, Silva et al., 2016)

#### 2.2.4 Insomnia Severity Index (ISI)

The ISI, developed by Bastien et al. (2001), assesses adolescents’ level of insomnia over the past two weeks. This seven-item self-report scale is rated on a five-point Likert-type scale ranging from 0 (*no problem*) to 4 (*very severe problem*). A total (sum of all seven items) score ranges from 0 to 28 with five sub-scores being 0–7 (absence of insomnia), 8–14 (sub-threshold insomnia), 15–21 (moderate insomnia), and 22–28 (severe insomnia) (Bastien et al., 2001). The Iranian version has an acceptable internal consistency (Cronbach’s α = 0.87; Ahorsu et al., 2020c, Yazdi et al., 2012).

#### 2.2.5 Pediatric Quality of Life Inventory™ 4.0 Short Form (PedsQL^TM^ 4.0 SF15)

The PedsQL^TM^ 4.0, developed by Varni et al. (2001), assesses quality of life among children using parallel child/adolescent self-reports and parent-reports. More specifically, the short-form 15-item version (Chan et al., 2005) was used in the present study. It is rated on a five-point Likert-type scale ranging from 0 (*never*) to 4 (*almost always*). The PedsQL™ 4.0 SF15 items can be calculated into four subscales and a total score for the child/adolescent self-report and parent-report. Scale scores are calculated as the sum of the items divided by the number of items answered. The scale has been validated among Iranians with Cronbach’s α of 0.82 and 0.84 for child self-report and parent proxy-report respectively (Pakpour, 2013).

### 2.3 Data analyses

Pearson’s correlations were first used to examine the relationships between the study’s variables (IGD, insomnia, depression, anxiety and stress, adolescent-reported quality of life, and parent-reported quality of life). Three mediation analyses were conducted to examine whether psychological distress (depression, anxiety, stress) was a significant mediator in the association between IGD and insomnia, IGD and adolescent-reported quality of life, and IGD and parent-reported quality of life. Furthermore, age, gender father’s and mother’s education were controlled for in these mediation models. The PROCESS macro for SPSS was used for the mediation analyses using model 4 and 10,000 bootstrapping resamples (Model 4, Process Macro) (Hayes, 2018). In addition, indirect effects were contrasted using Hayes’ macros (Preacher & Hayes, 2008).

## 3 Results

Table 1 shows the characteristics of the adolescents (N = 1512) with more than half being males (n = 853, 56.4%). On average, they spent 68.12 min/day (SD = 39.83) gaming online during weekends. Also, their mean IGD score (on the IGDS9-SF) was 19.07 (SD = 7.31). Their mean psychological distress scores (on the DASS) were 7.24 for depression (SD = 4.93), 8.46 for anxiety (SD = 5.64), and 6.87 for stress (SD = 5.11). Their adolescent-reported quality of life score (on the PedsQL^TM^ 4.0 SF15) was 74.38 (SD = 19.30) and parent-reported quality of life score (on the PedsQL^TM^ 4.0 SF15) of 69.61 (SD = 20.84). Their insomnia severity score (on the ISI) was 9.94 (SD = 5.59).

**Table 1:** Characteristics of the study participants (N = 1512).

	Mean and (±SD) or n (%)
Age (years)	15.51 (±2.75)
Gender (Males)	853 (56.4%)
Father’s educational years	6.33 (±3.55)
Mother’s educational years	7.68 (±3.86)
Average time spent playing Internet game during weekend (minutes/day)	68.12 (±39.83)
Internet Gaming Disorder Scale-Short Form	19.07 (±7.31)
Depression	7.24 (±4.93)
Anxiety	8.46 (±5.64)
Stress	6.87 (±5.11)
PedsQL^TM^ 4.0 SF15 (adolescent report)	74.38 (±19.30)
PedsQL^TM^ 4.0 SF15 (parent report)	69.61 (±20.84)
Insomnia Severity Index	9.94 (±5.59)

Table 2 shows the interrelationships between insomnia, depression, anxiety, stress, IGD, and quality of life (including both adolescent and parent reports). All the correlations were significant and in the anticipated direction (*p* < 0.05, absolute *r* range 0.19–0.71).

**Table 2:** Pearson correlation matrix of the variables of interest.

Variables	Insomnia	Depression	Anxiety	Stress	Gaming disorder	Quality of life (adolescent report)	Quality of life (parent report)
Insomnia^a^	—	0.33**	0.34**	0.24**	0.48**	−0.26**	−0.19**
Depression^b^		—	0.57**	0.28*	0.23**	−0.38**	−0.28**
Anxiety^c^			—	0.22**	0.20**	−0.33**	−0.22**
Stress^d^				—	0.21**	−0.40**	0.30**
Gaming disorder^e^					—	−0.35**	0.30**
Quality of life (adolescent report) f						—	0.71**
Quality of life (Parent report) g							—

* *p* < 0.05; ** *p* < 0.01.

a Assessed using Insomnia Severity Index (ISI).

b Assessed using Depression, Anxiety and Stress Scale (DASS-21).

c Assessed using Depression, Anxiety and Stress Scale (DASS-21).

d Assessed using Depression, Anxiety and Stress Scale (DASS-21).

e Assessed using Internet Gaming Disorder Scale-Short Form.

f Assessed using PedsQL^TM^ 4.0 SF15.

g Assessed using PedsQL^TM^ 4.0 SF15.

Table 3 shows that depression (unstandardized coefficient = 0.005; LLCI = 0.006; ULCI = 0.010), anxiety (unstandardized coefficient = 0.006; LLCI = 0.002; ULCI = 0.012), and stress (unstandardized coefficient = 0.003; LLCI = 0.001; ULCI = 0.007) were significant mediators in the association between IGD and insomnia. Therefore, the total indirect effect (0.014) was significant (LLCI = 0.007; ULCI = 0.023). Also, there were significant direct effects of IGD on the mediators and insomnia (unstandardized coefficient of 0.070; SE = 0.007; *p <* 0.001) with a significant total effect on insomnia (unstandardized coefficient of 0.084; SE = 0.007; *p <* 0.001).

**Table 3:** Models of the effect of internet gaming disorder on insomnia with depression, anxiety and stress as mediators.

	Unstand. Coeff.	SE or (Bootstrapping SE)	t-value or (Bootstrapping LLCI)	p-value or (Bootstrapping ULCI)
Total effect of internet gaming disorder on Insomnia	0.084	0.007	11.983	<0.001
Direct effect of internet gaming disorder on Insomnia	0.070	0.007	10.039	<0.001
Direct effect of internet gaming disorder on mediators				<0.001
Depression	0.217	0.041	5.277	<0.001
Anxiety	0.216	0.0473	4.576	<0.001
Stress	0.205	0.0425	4.828	<0.001
Indirect effect of internet gaming disorder on Insomnia				
Total indirect effect	0.014	(0.004)	(0.007)	(0.023)
Via depression	0.005	(0.003)	(0.006)	(0.010)
Via anxiety	0.006	(0.002)	(0.002)	(0.012)
Via stress	0.003	(0.002)	(0.001)	(0.007)
(C1)	−0.002	(0.003)	(−0.009)	(0.005)
(C2)	0.001	(0.003)	(−0.004)	(0.008)
(C3)	0.003	(0.003)	(−0.002)	(0.010)

Note: Age, gender father’s and mother’s education were adjusted for the model.

Unstand. Coeff. = unstandardized coefficient.

LLCI = lower limit in 95% confidence interval.

ULCI = upper limit in 95% confidence interval.

(C1): Depression vs. anxiety.

(C2): Depression vs. stress.

(C3): Anxiety vs. stress.

Table 4 shows that depression (unstandardized coefficient = -0.154; LLCI = -0.320; ULCI = -0.036), anxiety (unstandardized coefficient = -0.096; LLCI = -0.198; ULCI = -0.021) and stress (unstandardized coefficient = -0.243; LLCI = -0.422; ULCI = -0.104) were significant mediators in the association between IGD and adolescent-reported quality of life. Also, the total indirect effect (-0.493) was significant (LLCI = -0.817; ULCI = -0.223). Also, there were significant direct effects of IGD on the mediators and adolescent-reported quality of life (unstandardized coefficient of −0.789; SE = 0.154; *p <* 0.001), as well as a significant total effect on adolescent-reported quality of life (unstandardized coefficient of −1.281; SE = 0.164; *p <* 0.001). Examination of the pairwise contrasts of the indirect effects (C1: depression vs. anxiety) indicated that the specific indirect effect via depression was larger than the specific indirect effect via anxiety on adolescent-reported quality of life (LLCI = -0.222; ULCI = -0.080). Also, examination of the pairwise contrasts of the indirect effects (C3: anxiety vs. stress) indicated that the specific indirect effect via stress was larger than the specific indirect effect via anxiety on adolescent-reported quality of life (LLCI = 0.016; ULCI = 0.309).

**Table 4:** Models of the effect of internet gaming disorder on adolescent-reported quality of life with depression, anxiety and stress as mediators.

	Unstand. Coeff.	SE or (Bootstrapping SE)	t-value or (Bootstrapping LLCI)	p-value or (Bootstrapping ULCI)
Total effect of internet gaming disorder on Adolescent-reported quality of life	−1.281	0.164	−7.826	<0.001
Direct effect of internet gaming disorder on Adolescent-reported quality of life	−0.789	0.154	−5.118	<0.001
Direct effect of internet gaming disorder on mediators				<0.001
Depression	0.228	0.043	5.324	<0.001
Anxiety	0.223	0.049	4.530	<0.001
Stress	0.219	0.044	4.986	<0.001
Indirect effect of internet gaming disorder on Adolescent-reported quality of life				
Total indirect effect	−0.493	(0.150)	(−0.817)	(−0.223)
Via depression	−0.154	(0.071)	(−0.320)	(−0.036)
Via anxiety	−0.096	(0.046)	(−0.198)	(−0.021)
Via stress	−0.243	(0.080)	(−0.422)	(−0.104)
(C1)	−0.058	(0.075)	(−0.222)	(−0.080)
(C2)	0.089	(0.079)	(−0.059)	(0.250)
(C3)	0.147	(0.075)	(0.016)	(0.309)

Note: Age, gender father’s and mother’s education were adjusted for the model.

Unstand. Coeff. = unstandardized coefficient.

LLCI = lower limit in 95% confidence interval.

ULCI = upper limit in 95% confidence interval.

(C1): Depression vs. anxiety.

(C2): Depression vs. stress.

(C3): Anxiety vs. stress.

Table 5 shows that depression (unstandardized coefficient = -0.116; LLCI = -0.263; ULCI = -0.007) and stress (unstandardized coefficient = -0.208; LLCI = -0.375; ULCI = -0.080) were the significant mediators in the association between IGD and parent-reported quality of life but not anxiety (unstandardized coefficient = 0.051; LLCI = -0.163; ULCI = 0.041). Nonetheless, the total indirect effect (-0.374) was significant (LLCI = -0.639; ULCI = -0.154). Also, there were significant direct effects of IGD on the mediators and parent-reported quality of life (unstandardized coefficient of −0.886; SE = 0.171; *p <* 0.001) as well as a significant total effect on parent-reported quality of life (unstandardized coefficient of −1.261; SE = 0.172; *p <* 0.001). Examination of the pairwise contrasts of the indirect effects (C3: anxiety vs. stress) indicated that specific indirect effect via stress was larger than the specific indirect effect via anxiety on parent-reported quality of life (LLCI = 0.013; ULCI = 0.341).

**Table 5:** Models of the effect of internet gaming disorder on parent-reported quality of life with depression, anxiety and stress as mediators.

	Unstand. Coeff.	SE or (Bootstrapping SE)	t-value or (Bootstrapping LLCI)	p-value or (Bootstrapping ULCI)
Total effect of internet gaming disorder on Parent-reported quality of life	−1.261	0.172	−7.322	<0.001
Direct effect of internet gaming disorder on Parent-reported quality of life	−0.886	0.171	−5.185	<0.001
Direct effect of internet gaming disorder on mediators				<0.001
Depression	0.228	0.042	5.365	<0.001
Anxiety	0.224	0.049	4.596	<0.001
Stress	0.212	0.044	4.793	<0.001
Indirect effect of internet gaming disorder on Parent-reported quality of life				
Total indirect effect	−0.374	(0.124)	(−0.639)	(−0.154)
Via depression	−0.116	(0.067)	(−0.263)	(−0.007)
Via anxiety	0.051	(0.051)	(−0.163)	(0.041)
Via stress	−0.208	(0.076)	(−0.375)	(−0.080)
(C1)	−0.065	(0.092)	(-0.267)	(0.104)
(C2)	0.093	(0.089)	(-0.69)	(0.278)
(C3)	0.158	(0.083)	(0.013)	(0.341)

Note: Age, gender father’s and mother’s education were adjusted for the model.

Unstand. Coeff. = unstandardized coefficient.

LLCI = lower limit in 95% confidence interval.

ULCI = upper limit in 95% confidence interval.

(C1): Depression vs Anxiety.

(C2): Depression vs Stress.

(C3): Anxiety vs Stress.

## 4 Discussion

The present study examined the role of depression, anxiety, and stress in mediating the associations between internet gaming disorder (IGD) and health outcomes of insomnia and quality of life. The correlation results showed that there were positive relationships between IGD, insomnia, depression, anxiety, and stress with small to large effects. These findings indicate that as one variable increases so do the other variables and vice versa (Cohen, 1988, Cohen, 1992), and is similar to previous studies (Sosso and Kuss, 2018, Wong et al., 2020). Adolescent-reported quality of life was negatively associated with IGD, insomnia, depression, anxiety, and stress with small to medium effects, indicating that as one variable increases the other variable decreases and vice versa (Cohen, 1988, Cohen, 1992). This is similar to previous studies (Adib-Hajbaghery et al., 2015, Freire and Ferreira, 2018, Raknes et al., 2017, Wartberg et al., 2017). Similarly, parent-reported quality of life-related negatively with insomnia, depression, and anxiety but positively with stress and IGD with small to medium effects. Like the adolescent-reported quality of life, as one variable increases the other variable decreases and vice versa except for stress and IGD which increased while the parent-reported quality of life decreased and vice versa. Nonetheless, there was a positive relationship between adolescent-reported quality of life and parent-reported quality of life with large effect which indicates good inter-rater reliability.

The interrelationships between IGD, depression, anxiety, stress, and insomnia found in the Pearson correlations can be explained by the mediation analysis. The results of the mediation analysis showed that there were directly significant associations between (i) IGD and insomnia, (ii) IGD and the mediators (depression, anxiety, and stress), and (iii) mediators (depression, anxiety, and stress) and insomnia. Moreover, the mediating effect suggested an indirect effect of IGD on insomnia via depression, anxiety, and stress, with the strongest mediator effect being for anxiety, followed by depression, and finally stress. This suggests that IGD significantly influences depression and anxiety levels among adolescents and could possibly lead to a disorder as reported in previous studies (Andreassen, 2015, Chen et al., 2020b). Most online gamers get overly involved in online activities thereby spending most of their time with these activities at the expense of other significant areas of their life including their education, families, and/or offline social relationships which may result in psychological consequences such as anxiety, depression, and distress (Zaremohzzabieh et al., 2014). Also, online gaming may start out as a coping strategy for adolescents. However, adolescents may increasingly rely on gaming as a coping method. Consequently, they may be preoccupied with online activities and become socially withdrawn from the real world which may cause significant psychological and emotional distress when trying to stop at a later point (Andreassen, 2015, Chen et al., 2020b, Kraut et al., 1998, Yu and Shek, 2018, Zaremohzzabieh et al., 2014). It then becomes understandable why higher levels of IGD are associated with insomnia because individuals with addictive behaviors usually report higher levels of anxiety, depression, and stress (Chen et al., 2020a, Griffiths et al., 2017, Männikkö et al., 2020), and individuals with higher levels of these conditions find it more difficult to sleep properly (Poorebrahim et al., 2020, Wong et al., 2020). These findings support the assumption that psychological distress (depression, anxiety, and stress) is strong mediator in the association between IGD and insomnia. Therefore, it may be appropriate to monitor and/or educate adolescents on more adaptive ways (in terms of duration and frequency) of playing videogames (online or not) in order to prevent future sleep challenges.

Similarly, the mediation analysis further explained the initial inter-relationships between IGD, depression, anxiety, stress, and quality of life found in the correlational analyses. The mediation results showed that there was a significant direct association between (i) IGD and quality of life, (ii) IGD and mediators (depression, anxiety, and stress), and (iii) mediators (depression, anxiety, and stress) and quality of life. It was also observed that depression, anxiety, and stress (in total) mediated the association between IGD and quality of life with the strongest mediated effects appearing for stress (for both adolescent-reported and parent-reported quality of life), followed by depression (for both adolescent-reported and parent-reported quality of life respectively), and finally anxiety (for adolescent-reported quality of life). This indicates that IGD is indirectly associated with quality of life among adolescents via (at least) depression and stress. That is, IGD strongly influenced adolescents’ stress followed by depression levels (positively) which then influenced their quality of life (negatively). As aforementioned, IGD may contribute to psychological distress (Andreassen, 2015, Chen et al., 2020b). Together, with the current evidence showing the relationship between psychological distress and quality of life among adolescents (Bonsu et al., 2019, Hsu et al., 2014), the results of the mediation analyses confirm that psychological distress is a mediator in the association between IGD and quality of life. This further suggests that IGD may have multiple pathways in affecting adolescents’ quality of life. Therefore, family members and/or guardians need to pay special attention to how much time and how frequently their child invests in online gaming.

Furthermore, the use of adolescent reports for assessing quality of life alongside parental reports as a supplement have been more commonplace among recent quality of life studies. However, the present study found that adolescent and parent reports provided similar findings. Therefore, the present authors are confident in the associations found between quality of life and other variables. This is similar to previous studies that reported no significant differences between adolescent-reported and parent-reported quality of life (Lin et al., 2013a, Lin et al., 2013b, Su et al., 2013). Furthermore, depression (compared with anxiety) and stress (compared with anxiety) were found to largely account for specific indirect effects on quality of life.

In general, the findings suggest that depression, anxiety, and stress serve as strong mediators in the association between IGD, insomnia, and quality of life among adolescents during the COVID-19 pandemic. The findings also imply that there are multiple ways in which IGD becomes associated with insomnia and quality of life, and therefore it may be complicated dealing with challenges that arise from IGD. However, although the recent contemporary literature acknowledges the importance of online gaming during the COVID-19 pandemic period due to lockdowns and physical distancing (Amin et al., 2020, King et al., 2020, Ko and Yen, 2020), it may be prudent for children and their parents to monitor the amount of time invested in online gaming. Parents need to educate and monitor their children about being overly dependent on online activities including gaming. Parents should guide their children to utilize multiple different activities and/or adaptive coping strategies to deal with the challenges of COVID-19 pandemic.

### 4.1 Limitations

This study comprised adolescents aged between 13 and 18 years and so the findings may not be generalised to younger children or adults. Also, the government reactions and policies to control COVID-19 infection in Iran may be very different from other countries and therefore replication may be needed to more comprehensively understand how the variables used in the present study relate to countries. A cross-sectional design was utilized which, at best, provided only strong associations between variables of interest and so longitudinal or a randomized control trial studies are needed to examine causality effects and other parameters of IGD’s impact on adolescents’ quality of life. The data analyzed in the present study were nested and random effects due to the nesting feature were not been controlled for or assessed. Therefore, future study on the same topic should attempt to control for the random effects due to the nesting feature. For example, using multilevel mixed effects modeling may be a solution. Finally, mediating relationships (due to notions related to the sequence of causality) should ideally be examined utilizing longitudinal data. Given that the present study used a cross-sectional design, caution should be exercised when interpreting the mediation findings.

### 4.2 Conclusion

The present study confirmed the mediating effect of depression, anxiety, and stress on the associations between IGD and insomnia, adolescent-reported quality of life, and parent-reported quality of life. It was also found that IGD directly influenced insomnia and quality of life of adolescents with small to large significant relationships between all these variables. Therefore, this suggests that IGD is associated with different psychosocial outcomes with multiple pathways. The findings help inform researchers and clinicians on the mechanisms underlying the associations between IGD and quality of life among adolescents which will further research and help with how they educate and/or manage IGD among adolescents. Parents need to pay special attention to how much time and how frequently their children play videogames in general, as well as the pandemic period more specifically. Parents may also need to help their children deal with the psychological distress during the COVID-19 pandemic period.

The authors declare that they have no known competing financial interests or personal relationships that could have appeared to influence the work reported in this paper.

## 5 Role of funding sources

Funding for this study was provided by the Qazvin University of medical Science (QUMS), Qazvin, Iran. The funding sources did not have any significant influences on data collection, analyses, writing, or the decision to submit the manuscript for publication.

## CRediT authorship contribution statement

**Sara Fazeli:** Conceptualization, Data curation, Formal analysis, Resources, Writing - review & editing. **Isa Mohammadi Zeidi:** Conceptualization, Data curation, Writing - review & editing. **Chung-Ying Lin:** Conceptualization, Resources, Supervision, Validation, Visualization, Writing - original draft, Writing - review & editing. **Peyman Namdar:** Conceptualization, Data curation, Writing - review & editing. **Mark D. Griffiths:** Conceptualization, Supervision, Validation, Visualization, Writing - original draft, Writing - review & editing. **Daniel Kwasi Ahorsu:** Conceptualization, Resources, Supervision, Validation, Visualization, Writing - original draft, Writing - review & editing. **Amir Pakpour:** Conceptualization, Data curation, Formal analysis, Funding acquisition, Resources, Supervision, Validation, Visualization, Writing - original draft, Writing - review & editing.

## Declaration of Competing Interest

None.
